# Validation and method comparison for a point-of-care lateral flow assay measuring equine whole blood insulin concentrations

**DOI:** 10.1177/10406387221142288

**Published:** 2022-12-08

**Authors:** Emily H. Berryhill, Naomi S. Urbina, Sam Marton, William Vernau, Flavio H. Alonso

**Affiliations:** Departments of Medicine and Epidemiology, School of Veterinary Medicine, University of California–Davis, Davis, CA, USA; Fortis Life Sciences, San Diego, CA, USA; Fortis Life Sciences, San Diego, CA, USA; Pathology, Microbiology, and Immunology, School of Veterinary Medicine, University of California–Davis, Davis, CA, USA; Pathology, Microbiology, and Immunology, School of Veterinary Medicine, University of California–Davis, Davis, CA, USA; Department of Biomedical Sciences, School of Veterinary Medicine, Ross University, Saint Kitts, West Indies

**Keywords:** endocrinopathy, equine metabolic syndrome, hyperglycemia, insulin, laminitis

## Abstract

The Wellness Ready Test (WRT) is a lateral flow, stall-side assay that measures equine insulin in whole blood and requires validation before recommending clinical use. We evaluated intra- and inter-assay precision and linearity and compared the WRT with a radioimmunoassay (RIA). Tested concentrations ranged from <139 to >695 pmol/L (<20 to >100 μIU/mL). For 20 replicates at each insulin level, intra-assay CVs of the WRT for insulin were 13.3%, 12.9%, and 15.3% at low (139–278 pmol/L; 20–40 μIU/mL), intermediate (278–417 pmol/L; 40–60 μIU/mL), and high (>417 pmol/L; >60 μIU/mL) concentrations, respectively. For 10 replicates at each level (3 assay lots), inter-assay CVs were 15.9%, 11.0%, and 11.7%, respectively. In the weighted linear regression of 5 measured insulin concentrations against expected concentrations, *R*^2^ = 0.98, slope = 1.02, and y-intercept = 14.4 pmol/L (2.08 μIU/mL). The Spearman correlation coefficient (*r_s_*) was 0.90 (95% CI: 0.85–0.94) between the WRT and RIA; the WRT = f(RIA) Passing–Bablok regression yielded the fit, y = 1.005x + 24.3 pmol/L (3.50 μIU/mL). The WRT result averaged 10.4% higher than the RIA result, with targeted bias of 25.9, 26.1, and 26.7 pmol/L (3.74, 3.76, and 3.84 μIU/mL) for cutoffs used to diagnose insulin dysregulation of 312, 347, and 451 pmol/L (45, 50, and 65 μIU/mL). Assay clinical sensitivities, specificities, and accuracies determined at the 3 selected clinical cutoffs and using the RIA as gold standard were 87–95%, 92–96%, and 91–95%, respectively (*n* = 99 samples). Observed total error was 28.4–30.4%. The WRT had acceptable precision, excellent linearity, and good association with the RIA.

Insulin dysregulation is a key component of the equine metabolic syndrome (EMS) and often of equine pituitary pars intermedia dysfunction. Hyperglycemia and hyperinsulinemia can be exacerbated by other pathologic conditions, such as endotoxemia, as well as iatrogenically with either localized (e.g., intra-articular) or systemic corticosteroid use.^10,11^ Laminitis is one of the most severe and life-threatening consequences of hyperinsulinemia, with acute and chronic bouts commonly occurring in horses with endocrinopathies, with or without additional associated factors.^9^

Plasma insulin concentration measurement in horses is used to diagnose insulin dysregulation, assess laminitis risk, and gauge response to medical intervention once insulin dysregulation has been diagnosed. The Wellness Ready Test (WRT; Wellness Ready Labs) is a lateral flow assay (LFA) that is used as a point-of-care test (POCT) to measure whole blood insulin in horses. LFAs measure analytes in biological fluids by first exposing the analyte to a target antibody and then exposing the analyte–antibody complex to a test strip with a secondary antibody.^6^ The concentration of the analyte can be measured in a qualitative (e.g., human pregnancy test or SARS-CoV2 antigen tests) or quantitative (e.g., WRT) fashion. LFAs are desirable screening tools for medical conditions given their portability, low production cost, and rapid results (5–30 min). If a POCT is not available, equine samples are sent to reference laboratories for insulin analysis, which often results in added expense, delay in results and treatment initiation, and increased pre-analytical error and inaccuracy if plasma is not handled appropriately. Given ease of testing and immediacy of results, a readily available, validated POCT could dramatically increase the number of horses tested for insulin dysregulation, which could result in a larger number of horses receiving appropriate screening and treatment for potentially life-threatening conditions.

Our objective was to validate the WRT for use in measuring equine insulin in whole blood. Components of our study included test result repeatability (intra- and inter-assay precision), assessment of predicted sample concentrations (linearity), and comparison with a reference insulin assay. We hypothesized that insulin concentrations determined on whole blood with the WRT would show acceptable precision, accuracy, and linearity as determined by recommended quality assurance guidelines, and good association with the concentrations obtained using the reference method.

## Materials and methods

We divided our study into 4 components: intra-assay precision, inter-assay precision, linearity, and method comparison. Sample size was determined based on the American Society for Veterinary Clinical Pathology (ASVCP) quality assurance guidelines and previous POC assay validation studies.^3,9^ Whole blood for analysis was obtained from horses from the research herd at the University of California–Davis Center for Equine Health following Institutional Animal Care and Use Committee approval (protocol 20751). Horses were of varied signalment and were categorized based on anticipated plasma insulin concentrations utilizing data obtained previously. Whole blood was collected after ~12 h of fasting, normal feeding of a hay meal, or after an oral sugar test (OST) using 0.15–0.45 mL/kg of light corn syrup to obtain insulin concentrations spanning the working range of the assay.^8^

### Wellness Ready insulin test

**Figure 1.**
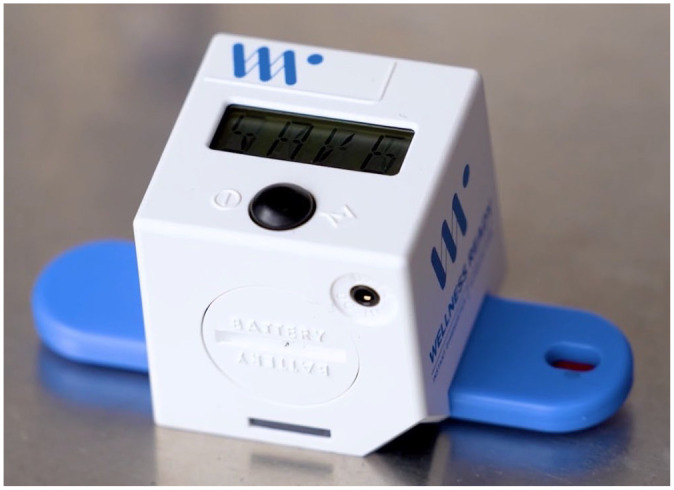
The Wellness Ready Test cube reader and lateral flow insulin assay kit. Photo courtesy of https://wellnessready.com/.

The WRT is a LFA, with a manufacturer-reported dynamic range of 139–695 pmol/L (20–100 μIU/mL). The WRT is a traditional sandwich immunoassay that utilizes an anti-porcine insulin antibody conjugated to a 40-nm gold reporter particle using a biotin–streptavidin interaction, and an anti-human insulin antibody adsorbed to a nitrocellulose membrane. The assay is designed to analyze whole blood; EDTA blood is first mixed with running buffer, then dropped onto a blood filter pad on the test device. As the sample flows laterally, the endogenous insulin binds to a gold particle–labeled detection antibody, and then this complex is captured by a stripe of another monoclonal antibody at the test line position. A secondary antibody at the control line position ensures that the test has run properly. Intensity of red color at the test line is proportional to the concentration of insulin in the sample. After a 15-min incubation, a portable reader (Fig. 1) converts this signal to a calculated concentration based on a programmed calibration curve established for each lot of test devices. Each lot is calibrated by the manufacturer at concentrations wider than the commercially reported dynamic range. We performed pilot studies to determine preliminary inter- and intra-assay precision before we conducted our method comparison study. For the purpose of assay validation, readers were programmed by the manufacturer to provide quantitative data both above and below the limits of the dynamic range published on the package insert.

### Sample processing and analysis

Whole blood samples were obtained from the jugular vein, stored in EDTA Vacutainer tubes (Becton Dickinson), and analyzed using the WRT within ~2 h of sample collection, according to test kit instructions. Raw signal value and calculated insulin concentrations from the readers were recorded. Additional whole blood in EDTA was refrigerated at 4°C for up to 4 h before centrifugation (1,507 × *g*, 10 min, 20°C), and plasma was aliquoted into cryovials. Samples were frozen at −20°C for up 4 d before being transferred to a −80°C freezer for long-term storage.

### Intra- and inter-assay precision

Whole blood was obtained from 3 horses with anticipated insulin concentrations of 139–278 pmol/L (20–40 μIU/mL; low), >278–417 pmol/L (>40–60 μIU/mL; intermediate), and >417 pmol/L (>60 μIU/mL; high). For each blood sample, 20 replicates were analyzed using kits from 1 lot number and a single WRT reader to determine intra-assay precision. Ten replicates were also analyzed using kits from a second and third lot number and up to 13 different WRT readers. To determine inter-assay precision, 10 replicates were randomly selected from the first lot (used for intra-assay precision) so that there was an equal number of replicates across all 3 lots. Replicates from each lot were analyzed in batches of 2–4 at a time to minimize the effects of time on insulin concentrations (i.e., 2–4 replicates from lot A, lot B, and lot C were analyzed at the same time; then 2–4 more replicates from each lot were analyzed, etc.).

### Linearity

Whole blood was obtained from a horse with an insulin concentration <139 pmol/L (<20 μIU/mL; low insulin concentration, representing lower reportable limit of the dynamic range) and a horse with an insulin concentration approaching 695 pmol/L (100 μIU/mL; high insulin, representing upper reportable limit of dynamic range). Dilutions were created with 1) 100% high-insulin blood, 2) 75% high-insulin blood, 25% low-insulin blood, 3) 50% high- and 50% low-insulin blood, 4) 25% high- and 75% low-insulin blood, and 5) 100% low-insulin blood. Three replicates of each dilution were analyzed.

### Method comparison

Ninety-nine blood samples were obtained from 51 horses, with insulin concentrations ranging from <139 pmol/L (<20 μIU/mL) to >695 pmol/L (>100 μIU/mL). Whole blood samples were analyzed in duplicate and statistically analyzed as both single measurements and as a $\bar{x}$ of the 2 measurements. The WRT package insert does not specify testing in duplicate; we tested duplicates to assess assay precision and to match the protocol used for the reference method. For the reference method, plasma aliquots from the same blood samples were shipped overnight on ice to the Cornell University Animal Health Diagnostic Center (AHDC; Ithaca, NY, USA) for analysis using a competitive human insulin–specific radioimmunoassay (RIA; EMD Millipore) utilizing a guinea pig anti-human insulin–specific antibody, internally validated at the AHDC for use in equids. The $\bar{x}$ of 2 measurements from the plasma sample submitted to the AHDC was used in analysis, as is standard for RIAs. The human insulin RIA was selected as the reference assay given its common use in equine clinical practice, as well as its inclusion in the most recent guidelines on EMS (https://sites.tufts.edu/equineendogroup/files/2022/10/EMS-EEG-Recommendations-2022.pdf).

### Statistics

Statistical analysis was performed (Excel v.2202, Microsoft; Analyse-it v.5.92, an add-in for Excel; R v.4.0.3, https://www.r-project.org/). Insulin concentrations of 313 pmol/L (45 μIU/mL), 347 pmol/L (50 μIU/mL), and 451 pmol/L (65 μIU/mL) were selected as cutoffs for determining clinical sensitivity, specificity, and diagnostic accuracy because they are the recommended cutoffs for diagnosing insulin dysregulation after a 0.15-mL/kg OST, basal (no dynamic testing) analysis, and high-dose 0.45-mL/kg OST, respectively, when insulin is measured by RIA (https://sites.tufts.edu/equineendogroup/files/2022/10/EMS-EEG-Recommendations-2022.pdf).

Intra- and inter-assay precision were estimated by determining the $\bar{x}$ , SD, and CV ([SD/ $\bar{x}$ ] × 100%) across replicates and lots, respectively. Linearity was assessed by plotting the $\bar{x}$ measured concentration of the 3 replicates of each sample in the dilution series against the expected concentrations. The results were further analyzed by weighted linear regression (weighted by the inverse variance across the replicates of each sample) to determine the slope, intercept, and goodness-of-fit of the model (*R*^2^).

Bias of the WRT versus the RIA was assessed using Passing–Bablok linear regression, Spearman correlation, and Bland–Altman plots in which the means of the duplicate whole blood measurements were used. Targeted bias at the clinical cutoffs was calculated by the formula: (calculated concentration – clinical cutoff value)/clinical cutoff value × 100%, in which the calculated concentration was the WRT concentration derived from the Passing–Bablok regression fit at the clinical cutoff value. Clinical sensitivity, specificity, and overall accuracy for the WRT were determined for whole blood insulin concentrations, using results from the RIA as the gold standard at the same cutoff concentrations. Method comparison results for the WRT were evaluated using both the $\bar{x}$ of the duplicates and keeping the duplicates separate. CIs around the point estimates of sensitivity, specificity, and overall accuracy were calculated by the normal approximation (Wald interval) for the $\bar{x}$ of the duplicates and by a method for clustered binary data for the duplicates.^13^

Observed total error (TEo) was calculated with the formula: |bias| (%) + 2CV, at the 3 clinical cutoffs using the respective targeted bias from the Passing–Bablok regression and the inter-assay CV for the intermediate-insulin category (Table 1).^4^

**Table 1.** Intra- and inter-assay precision of the Wellness Ready Test for equine insulin using whole blood. Twenty replicates for each insulin category (low, intermediate, and high) were performed to determine intra-assay precision. Ten replicates were performed for each of 2 additional lot numbers and were combined with 10 randomly selected replicates from the first lot to determine inter-assay precision for each insulin category.

Sample	Intra-assay (*n* = 20)			Inter-assay (*m* = 3)		
	$\bar{x}$ concentration, pmol/L (μIU/mL)	SD	CV, %	$\bar{x}$ concentration, pmol/L (μIU/mL)	SD	CV, %
Low, 139–278 pmol/L (20–40 μIU/mL)	178 (25.7)	23.6 (3.40)	13.3	170 (24.5)	24.5 (3.90)	15.9
Medium, 278–417 pmol/L (40–60 μIU/mL)	327 (47.1)	42.2 (6.07)	12.9	313 (45.1)	34.5 (4.97)	11.0
High, >417 pmol/L (>60 μIU/mL)	527 (75.9)	80.7 (11.6)	15.3	511 (73.5)	59.6 (8.57)	11.7

*m* = number of assay lots tested.

## Results

### Intra- and inter-assay precision

For the 278–417 pmol/L (40–60 μIU/mL) group, 19 replicates were performed for intra-assay precision because of 1 test failure, and 11 replicates were performed for each of the 2 additional kit lots to determine inter-assay precision (Table 1). Intra-assay CVs for low, intermediate, and high insulin concentrations were 13.3%, 12.9%, and 15.3%, respectively. Inter-assay CVs for low, intermediate, and high insulin concentrations were 15.9%, 11.0%, and 11.7%, respectively.

### Linearity

**Figure 2.**
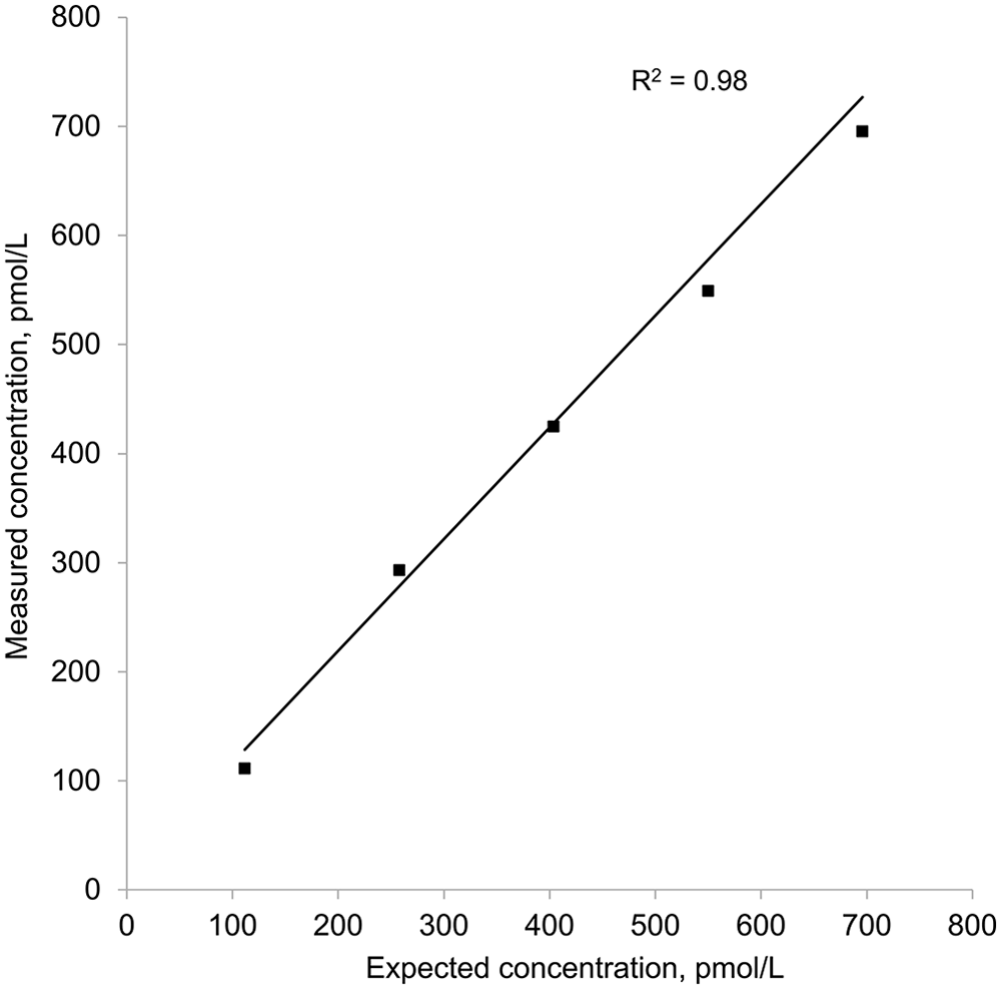
Weighted linear regression of 5 equine insulin concentrations measured with the Wellness Ready Test against calculated expected insulin concentrations. Weights were calculated as the inverse variance of the 3 replicates performed for each sample. Measured concentration = (1.02 × expected concentration) + 14.4.

Four replicates were performed at the high insulin concentration, with 1 replicate removed as an outlier (Dixon test, *p* = 0.047; Table 2, Fig. 2).

**Table 2.** Expected and measured insulin concentrations reported by the Wellness Ready Test for equine insulin in whole blood, with CV and bias between the expected and measured $\bar{x}$ concentrations to assess assay linearity. Dilutions of whole blood were made by adding blood with low insulin concentration to blood with high insulin concentration. The expected concentrations were derived mathematically.

Sample	Sample preparation	Measured concentration, pmol/L (μIU/mL)					Expected concentration, pmol/L (μIU/mL)	Bias,^†^ %
		Rep 1	Rep 2	Rep 3	$\bar{x}$	CV,* %		
1	High	837 (121)	629 (90.5)	622 (89.5)	696 (100)	17.6	696 (100)	0
2	75% high + 25% low	606 (87.2)	542 (78.1)	500 (72.0)	549 (79.1)	9.7	550 (79.2)	0
3	50% high + 50% low	479 (69.0)	402 (57.9)	394 (56.8)	425 (61.2)	11.0	404 (58.2)	5
4	25% high + 75% low	293 (42.2)	274 (39.5)	313 (45.1)	294 (42.3)	6.6	258 (37.1)	14
5	Low	127 (18.3)	82.6 (11.9)	125 (18.0)	112 (16.1)	22.5	112 (16.1)	0

Rep = replicate.

* CV = (SD of the 3 replicate concentrations/ $\bar{x}$ ) × 100%.

† Bias = ( $\bar{x}$ measured concentration – expected concentration)/expected concentration) × 100%.

### Method comparison

**Figure 3.**
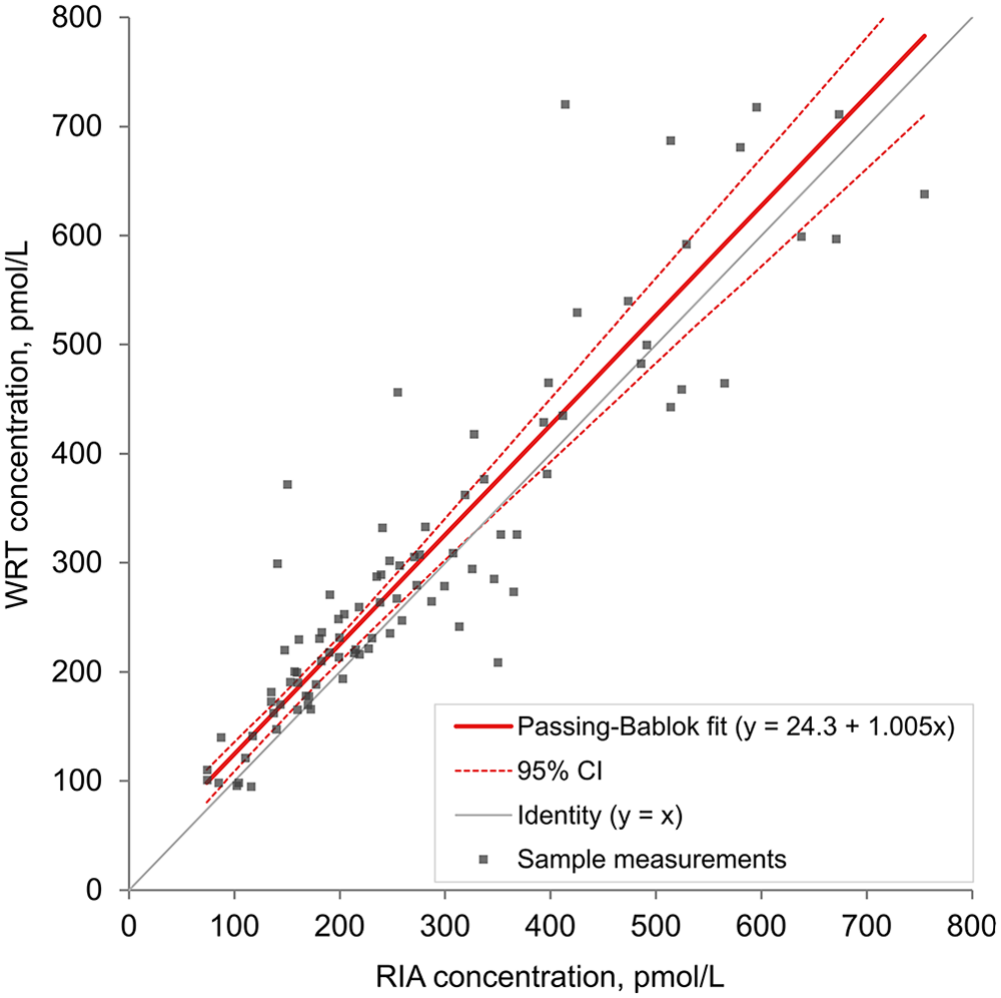
Passing–Bablok linear regression of equine insulin concentrations (*n* = 91) analyzed with a lateral flow assay (Wellness Ready Test, WRT) using whole blood and with a human insulin radioimmunoassay (RIA) using plasma. The $\bar{x}$ concentrations of whole blood and plasma replicates were used in the calculation. The y-intercept = 24.3 (95% CI: 1.2–41.5) and slope = 1.005 (95% CI: 0.90–1.01).

**Figure 4.**
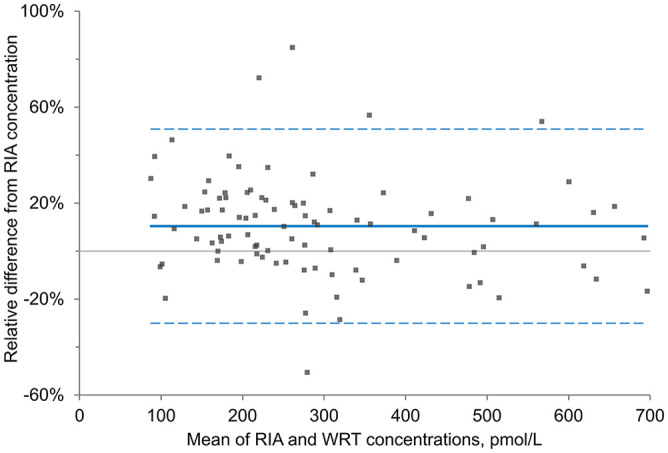
Bland–Altman plot of the percent difference between equine insulin concentrations for 91 paired samples determined with a lateral flow assay (Wellness Ready Test, WRT) using whole blood and a human insulin radioimmunoassay (RIA) using plasma. $\bar{x}$ relative difference (solid line) = 10.4%; 95% limit of agreement (dashed lines) = −30.1 to 50.9%.

Eight samples with high raw signal readings (in horses that were given an OST) were excluded from the regression, correlation, and Bland–Altman analyses, given a non-numeric WRT result because the sample concentration was beyond the limit of the calibration curve of the assay. The number of horses with insulin concentrations of 139–417 pmol/L (20–60 μIU/mL) was similar between the RIA and WRT, with mildly increased divergence as concentrations approached the limits of the working range of the assay (Table 3). The Spearman correlation showed good association between the WRT and RIA, with a correlation coefficient (*r_s_*) of 0.90 (95% CI: 0.85–0.94). The WRT = f(RIA) Passing–Bablok regression equation was y = 1.005x + 24.3 pmol/L (3.50 μIU/mL; Fig. 3). The targeted biases at the designated specific cutoffs were 25.9 pmol/L (95% CI: 1.29–41.8; 3.74 μIU/mL, 95% CI: 0.19–6.02), 26.1 pmol/L (95% CI: −2.08 to 45.1; 3.76 μIU/mL, 95% CI: −0.30 to 6.49), and 26.7 pmol/L (95% CI: −12.6 to 55.8; 3.84 μIU/mL, 95% CI: −1.82 to 8.03) at 313, 347, and 451 pmol/L (45, 50, and 65 μIU/mL), respectively. The targeted biases were 8.3%, 7.5%, and 5.9% at 313, 347, and 451 pmol/L (45, 50, and 65 μIU/mL), respectively. Whole blood insulin concentrations measured by the WRT averaged 10.4% (95% CI: 6.09–14.7) higher than plasma insulin concentrations obtained from the RIA when assessed by Bland–Altman analysis (Fig. 4). The clinical sensitivity, specificity, and overall accuracy for whole blood measurements of the WRT using a cutoff of 313 pmol/L (45 μIU/mL) were 87%, 92–93%, and 90–91%, respectively. The clinical sensitivity, specificity, and overall accuracy were 88%, 92–93%, and 90–91%, using a cutoff of 347 pmol/L (50 μIU/mL), and 91–95%, 95–96%, and 95% using a cutoff of 451 pmol/L (65 μIU/mL; (Table 4, Suppl. Table 1). The slight range in clinical parameters occurred because of minor differences when sensitivity and specificity were calculated using the $\bar{x}$ of the duplicates as a single measurement or as separate measurements.

**Table 3.** Distributions of insulin concentrations assessed with the Millipore human radioimmunoassay (RIA) as the reference assay and the Wellness Ready Test (WRT).

	Concentration range, pmol/L (μIU/mL)					Total, *n*
	<139 (<20), *n*	139–278 (20–40), *n*	>278–417 (>40–60), *n*	>417–695 (>60–100), *n*	>695 (>100), *n*	
RIA	12	45	19	14	9	99
Whole blood WRT	7	43	20	18	11	99

**Table 4.** Sensitivities, specificities, and overall accuracy for the Wellness Ready Test for equine insulin utilizing whole blood in comparison to the Millipore human radioimmunoassay (RIA) as the reference assay. Calculations were performed using the $\bar{x}$ of duplicate tests as a single measurement and also as separate measurements. Results are expressed as point estimates (%; 95% CI).

Cutoff, pmol/L (µIU/mL)	Sample	Sensitivity*	Specificity^†^	Overall accuracy^‡^	No. of samples
313 (45)	$\bar{x}$ of Rep 1 & 2	87 (76–98)	93 (87–100)	91 (85–97)	99
	Rep 1 & 2 separate	87 (77–97)	92 (86–97)	90 (85–95)	198
347 (50)	$\bar{x}$ of Rep 1 & 2	88 (76–99)	93 (86–99)	91 (85–97)	99
	Rep 1 & 2 separate	88 (76–99)	92 (86–98)	90 (85–96)	198
451 (65)	$\bar{x}$ of Rep 1 & 2	95 (87–100)	95 (90–100)	95 (91–99)	99
	Rep 1 & 2 separate	91 (83–99)	96 (92–100)	95 (91–99)	198

Rep = replicate.

* Sensitivity = true positives/(true positives + false negatives) × 100%.

† Specificity = true negatives/(true negatives + false positives) × 100%.

‡ Accuracy = (true positives + true negatives)/(true positives + true negatives + false positives + false negatives).

### Observed total error

The calculated TEo at the clinical cutoffs of 313 pmol/L (45 μIU/mL), 347 pmol/L (50 μIU/mL), and 451 pmol/L (65 μIU/mL) were 30.4%, 29.6%, and 28.0%, respectively (Table 5).

**Table 5.** Observed total error (TE_O_) calculated from the inter-assay CV at the intermediate-insulin concentration category (see Table 1) and the targeted bias at the 3 clinical cutoff insulin concentrations derived from the Passing–Bablok regression of the method comparison.

Clinical cutoff value, pmol/L (μIU/mL)	CV, %	Targeted bias, %	TEo,* %
313 (45)	11.0	8.3	30.4
347 (50)	11.0	7.5	29.6
451 (65)	11.0	5.9	28.0

* TEo = |bias| (%) + 2CV.

## Discussion

Overall, the analytical precision and linearity of the WRT were acceptable, and results showed good association with plasma insulin concentrations measured with the reference RIA. Intra-assay precision was good for low and intermediate insulin concentrations, with the CV within guidelines recommended for bioanalytical method validation of <15%, and just outside of the recommendations for high insulin concentrations, with a CV of 15.3% (https://www.ema.europa.eu/en/documents/scientific-guideline/guideline-bioanalytical-method-validation_en.pdf).

The CV was lowest in the intermediate-insulin concentration category (278–417 pmol/L; 40–60 μIU/mL), which is clinically important because this range is most critical in determining if horses have insulin dysregulation, particularly when performing an OST. The CV for intra-assay precision was highest in the high-insulin category, with increased SD as the $\bar{x}$ insulin concentration approached the upper limits of the reported dynamic range of the WRT. Inter-assay precision was considered adequate, with CVs of 11.0% for intermediate concentrations and up to 15.9% for low concentrations. In clinical laboratory settings, it is common for the CV to increase as the analyte concentration approaches the limit of the dynamic range of an assay; the higher inter-assay CV of 15.9% for the low insulin concentration is consistent with this scenario.^5^ As a comparison, $\bar{x}$ intra- and inter-assay CV for the human insulin RIA are reported to be 3.9% and 5.3%, respectively.^2^ Intra-assay CVs at each insulin level in our study were also higher than those reported for an equine insulin ELISA (4.6% at low insulin concentrations and 1.9% at intermediate concentrations), as were the inter-assay CVs (7.3% for low, 4.8% for high insulin concentrations for the ELISA).^10^

Inherent tester variability is one possible explanation for the higher intra- and inter-assay CVs for the WRT. Although 4 well-trained people performed the test procedure, there is always the possibility that small volume variations from individual bulb pipettes or running buffer might have resulted in random discrepancies across tests given that they were done by hand rather than automated. Because this product is designed for stall-side use, it was important to perform the assays by hand, just as they would be performed in real-life scenarios to provide clinically applicable data. As a comparison, intra-assay CVs for an equine serum amyloid A POCT, also performed by hand, were 15%, 18%, and 13% for low, medium, and high concentrations (*n* = 20 each), respectively.^7^ Additionally, hemoconcentration was evaluated by the manufacturer as a possible contributing factor to intra-assay variation and was found to be nonsignificant (Urbina N, pers. comm., Nov 2021). A limitation of calculating inter-assay CVs is that, given manufacturing limitations, 10 additional replicates of each of 2 lots were performed, whereas ASVCP guidelines recommend 20 replicates. The resulting inter-assay CVs could have been biased as a result. That said, results are still largely within the recommended inter-assay CV guidelines for assay performance.

The WRT had excellent linearity for measured versus calculated insulin concentrations, with a slope of nearly 1 and *R*^2^ of 0.98. Thus, there can be a high degree of confidence that, within the reportable dynamic range, insulin concentrations obtained using the assay should correlate well with expected concentrations in a linear manner over the working range of the assay. One limitation of our study was the lack of a commercial equine insulin standard ideally used to create dilutions. Without the standard, the next best approach was to obtain a whole blood sample with insulin concentrations slightly below the lower limit of the dynamic range and use this to dilute blood with a high insulin concentration. The assay linearity was excellent utilizing this acceptable compromise approach.^9^

Spearman correlation analysis showed that the RIA and WRT methods are strongly associated, with a coefficient close to 1 (*r_s_* = 0.90). Similarly, Passing–Bablok regression indicated a strong linear relationship between the 2 assays across the insulin concentration range, with a slope of 1.005 and a small y-intercept. This result is similar to, but slightly less than, correlations found between the Millipore porcine RIA, an ELISA, and a chemiluminescence immunoassay (CLIA), in which the Spearman correlation coefficient was 0.992 for the RIA and ELISA and 0.997 for the RIA and CLIA.^12^ However, a limitation of the WRT is the apparent dispersion of data points around the regression line, particularly at higher concentrations.. This is in contrast to comparisons of the ELISA and RIA, ELISA and CLIA, and CLIA and RIA, in which there was less dispersion around the regression lines across sampling points.

In the Bland–Altman plot, the $\bar{x}$ relative difference was 10.4% for the WRT across the range of insulin concentrations tested, compared to the same plasma sample analyzed with the RIA. Additionally, the bias between the 2 assays as determined by Passing–Bablok regression was constant at ~24.3 pmol/L (~3.50 μIU/mL) over the range of observed concentrations; the targeted bias at specific insulin cutoff concentrations of interest (313, 347, and 451 pmol/L; 45, 50, and 65 μIU/mL) was 25.9–26.7 pmol/L (3.74–3.84 μIU/mL). This bias indicates, for example, that an insulin concentration of 347 pmol/L (50 μIU/mL) measured by RIA would be ~373 pmol/L (53.7 μIU/mL) measured by the WRT. Given that different types of insulin assays are known to measure insulin concentrations with variable degrees of bias, this result is not surprising.^1,12^

Assay-specific RIs facilitate the use of validated assays. The clinical significance of the difference in assay results depends on where the patient’s insulin concentration falls in relationship to the established clinical cutoffs for insulin dysregulation. For example, if a horse’s insulin concentration is 139 pmol/L (20 μIU/mL) or 660 pmol/L (95 μIU/mL), a 10% difference in either direction will not likely change clinical decision-making. However, an insulin concentration of 313 pmol/L (45 μIU/mL) with the WRT, particularly after a 0.15-mL/kg OST, could be more challenging to interpret in relation to the RIA and established guidelines that use RIA RIs. Knowing the bias at the specific cutoffs is helpful in clarifying interpretation of the WRT results with reference to expected RIA results, and a $\bar{x}$ difference of <27.8 pmol/L (4 μIU/mL, which would incorporate the bias at the selected cutoffs) is unlikely to change decision-making, particularly when interpreted in context with the patient’s phenotype and clinical presentation. That said, until appropriate RIs are established for the WRT, there may be utility in submitting additional plasma samples for RIA analysis if the whole blood WRT result falls at a decision-changing cutoff. Additionally, the upper limit of the dynamic range of the WRT (695 pmol/L; 100 μIU/mL) is sufficient for diagnosing IR but may be insufficient for monitoring horses with persistently severe elevations in insulin concentrations. For example, a decrease from a baseline insulin concentration of 2,432 pmol/L (350 μIU/mL) to 1,390 pmol/L (200 μIU/mL) as a response to treatment would be clinically significant but would not be detectable utilizing the WRT because both the baseline and post-treatment insulins would read as >695 pmol/L (100 μIU/mL); additional methodologies would be needed to appropriately monitor response.

When using the RIA as the reference comparison, the WRT had a moderate-to-high sensitivity of 87% and 88% at insulin cutoff concentrations of 313 and 347 pmol/L (45 and 50 μIU/mL), respectively. Specificity was higher at 92–93% for the same cutoffs. As the insulin cutoff increased to 451 pmol/L (65 μIU/mL), the sensitivity of the WRT increased to as high as 95%, with specificity of 96%. The overall accuracy of the test also increased from 90–91% at cutoffs of 313 and 347 pmol/L (45 and 50 μIU/mL) to 95% at a cutoff of 451 pmol/L (65 μIU/mL). As the insulin concentration cutoff increased to 65 μIU/mL, the false-negative rate decreased to as low as 5% when the $\bar{x}$ of the 2 replicates was used in the analysis. Specificity also increased up to 95% as the insulin concentration increased when the $\bar{x}$ of the 2 replicates was used, as did overall accuracy. In summary, versus the RIA, at insulin cutoffs of 313 and 347 pmol/L (45 and 50 μIU/mL), there was a 7–8% chance of over-diagnosing and a 13% chance of under-diagnosing insulin dysregulation. Horses with basal insulin concentrations >451 pmol/L (>65 μIU/mL) or those induced with a high-dose OST were more likely (95%) to be diagnosed with true insulin dysregulation, which is expected given the increased accuracy of the WRT at higher concentrations. This is clinically relevant because horses with elevated insulin concentrations outside the equivocal zone of <50 μIU/mL have a high likelihood of being diagnosed accurately using the WRT (using the RIA as the gold standard), allowing appropriate management to be instituted quickly. Additionally, we evaluated clinical sensitivity and specificity by using both the $\bar{x}$ of the duplicates and keeping duplicates separate. Overall, there was minimal change in sensitivities, specificities, or accuracies when the duplicates were treated differently, with the most significant difference being an increase in sensitivity from 91% to 95% at the cutoff of 451 pmol/L (65 μIU/mL) when the $\bar{x}$ of duplicates was used. It is reasonable to perform one replicate of the WRT in a clinical setting; however, duplicate testing could be considered around the 451 pmol/L (65 μIU/mL) cutoff if increased sensitivity is desired.

Total allowable error (TEa) is defined as a “quality goal that sets a limit for combined imprecision (random error) and bias (inaccuracy, or systemic error) that is tolerable in a single measurement to ensure clinical usefulness.”^7^ The cutoffs for TEa vary, depending on the analyte and the species. TEa is a concept, as opposed to a measurement such as TEo. Equations to calculate TEo include [|bias| (%) + 2CV] or [|bias| (units of test) + 2SD].^4^ A method can be considered as a candidate for use when TEo <TEa. To date, there are no published consensus TEa goals for veterinary endocrinology. Reported human insulin TEa goals include 20.8–34.7 pmol/L (3–5 μIU/mL) and 25–32.9% (https://datainnovations.com/allowable-total-error-table). The TEo of the WRT, based on the targeted bias at the 3 clinical cutoffs and the inter-assay CV for the intermediate-insulin category, were all within the TEa parameters reported for human insulin assays. However, additional research is required to determine the appropriate TEa of equine insulin assays, and the TEo of other equine insulin assays, prior to making recommendations.

For both the linearity and the method comparison portions of our study, insulin concentrations above and below the reported dynamic range of the WRT were recorded. For our purposes, the readers were programmed to provide quantitative data outside the bounds of what is available commercially (i.e., for concentrations <139 pmol/L [<20 μIU/mL] and >695 pmol/L [>100 μIU/mL], clinicians will see a “<20 μIU/mL” or “>100 μIU/mL,” respectively; our study group obtained numerical concentrations below and above these concentrations). Internal assay calibration extends above and below the dynamic range of the assay. Additionally, given the strong association between WRT and RIA results determined in our method comparison study, we had confidence that the quantitative data outside the commercially reported dynamic range were sufficiently accurate to reflect linearity appropriately across the range of concentrations evaluated. However, plasma samples from the linearity study were not submitted for RIA, which could have been performed to further strengthen the linearity data.

An important limitation in our study is use of the RIA as the gold standard. Using high-performance liquid chromatography–mass spectrometry (HPLC-MS) as the ideal gold standard for which to compare WRT measured insulin concentrations would provide the most robust measurement of accuracy; however, HPLC-MS was not available to us. We selected the RIA as the gold standard method given its common use in clinical and research settings, and its reference in current equine endocrinopathy guidelines. The RIA has been validated internally at the laboratory that performs the assay; however, peer-reviewed validation has not been performed, which is another limitation. Also, we had decreasing sample numbers with increasing insulin concentrations. Despite having preliminary data on expected insulin concentrations in this group of research horses, inherent variability in basal and OST insulin concentrations made it challenging to achieve equal distribution of insulin concentrations across a wide range. In addition, the sample concentration was beyond the upper limit of the reported dynamic range of the WRT in several samples of whole blood, further decreasing the sample number at these high concentrations for some of the statistical analyses.

The predictive indices of the WRT for use in the diagnosis of insulin dysregulation in equids will require comprehensive clinicopathologic testing and clinical evaluations in a large cohort of horses.

## Supplemental Material

sj-pdf-1-vdi-10.1177_10406387221142288 – Supplemental material for Validation and method comparison for a point-of-care lateral flow assay measuring equine whole blood insulin concentrations Click here for additional data file. Supplemental material, sj-pdf-1-vdi-10.1177_10406387221142288 for Validation and method comparison for a point-of-care lateral flow assay measuring equine whole blood insulin concentrations by Emily H. Berryhill, Naomi S. Urbina, Sam Marton, William Vernau and Flavio H. Alonso in Journal of Veterinary Diagnostic Investigation
