# Supplementary material for: Validation and method comparison for a point-of-care lateral flow assay measuring equine whole blood insulin concentrations
Source: J Vet Diagn Invest. 2022 Dec 8;35(2):124–31. doi: 10.1177/10406387221142288 (PMC9999392; doi:10.1177/10406387221142288)
Supplement: sj-pdf-1-vdi-10.1177_10406387221142288 – Supplemental material for Validation and method comparison for a point-of-care lateral flow assay measuring equine whole blood insulin concentrations [file sj-pdf-1-vdi-10.1177_10406387221142288.pdf]

Berryhill EH, et al. Validation and method comparison for a point-of-care lateral flow assay measuring equine whole blood insulin concentrations

Supplemental Table 1. Comparison of the Wellness Ready Test (WRT) lateral flow assay and a reference radioimmunoassay (RIA) measuring equine insulin concentrations. The table shows the number of samples classified correctly and incorrectly at each of 3 insulin cutoff concentrations (313, 347, and 451 pmol/L [45, 50, and 65 µIU/mL]), using the RIA as the gold standard. Results are shown using both the x̄ of 2 replicates and keeping the replicates separate for the WRT.

	x̄ of Rep 1 & 2		
	WRT ≤ 45	WRT > 45	Total
RIA ≤ 45	57	4	61
RIA > 45	5	33	38
Total	62	37	99
	Rep 1 & 2 separate		
	WRT ≤ 45	WRT > 45	Total
RIA ≤ 45	112	10	122
RIA > 45	10	66	76
Total	122	76	198
	x̄ of Rep 1 & 2		
	WRT ≤ 50	WRT > 50	Total
RIA ≤ 50	62	5	67
RIA > 50	4	28	32
Total	66	33	99
	Rep 1 & 2 separate		
	WRT ≤ 50	WRT > 50	Total
RIA ≤ 50	123	11	134
RIA > 50	8	56	64
Total	131	67	198
	x̄ of Rep 1 & 2		
	WRT ≤ 65	WRT > 65	Total
RIA ≤ 65	73	4	77
RIA > 65	1	21	22

Total	74	25	99

	Rep 1 & 2 separate		
	WRT ≤ 65	WRT > 65	Total
RIA ≤ 65	148	6	154
RIA > 65	4	40	44
Total	152	46	198

Rep = replicate.
